# Repurposing doxycycline for synucleinopathies: remodelling of α-synuclein oligomers towards non-toxic parallel beta-sheet structured species

**DOI:** 10.1038/srep41755

**Published:** 2017-02-03

**Authors:** Florencia González-Lizárraga, Sergio B. Socías, César L. Ávila, Clarisa M. Torres-Bugeau, Leandro R. S. Barbosa, Andres Binolfi, Julia E. Sepúlveda-Díaz, Elaine Del-Bel, Claudio O. Fernandez, Dulce Papy-Garcia, Rosangela Itri, Rita Raisman-Vozari, Rosana N. Chehín

**Affiliations:** 1 Instituto Superior de Investigaciones Biológicas (INSIBIO), CCT-Tucumán and Instituto de Química Biológica Dr Bernabé Bloj (CONICET-UNT), Chacabuco 461 (T4000ILI) Tucumán, Argentina; 2 Institut National De La Santé Et De La Recherche Médicale, U 1127, CNRS, Unité Mixte De Recherche (UMR) 7225, Sorbonne Universités, UPMC Univ Paris 06, UMR S 1127, Institut Du Cerveau Et De La Moelle Epinière, ICM, Paris, France; 3 Instituto de Física da Universidade de São Paulo – IFUSP, Rua do Matão, Travessa R, 187, São Paulo, Brazil; 4 Max Planck Laboratory for Structural Biology, Chemistry and Molecular Biophysics of Rosario (MPLbioR, UNR-MPIbpC) and Instituto de Investigaciones para el Descubrimiento de Fármacos de Rosario (IIDEFAR, UNR-CONICET), Universidad Nacional de Rosario, Ocampo y Esmeralda, S2002LRK Rosario, Argentina; 5 Department of Morphology, Physiology and Stomatology, Faculty of Odontology of Ribeirão Preto, University of São Paulo, Brazil, Center of Interdisciplinary Research on Applied Neurosciences (NAPNA), University of São Paulo, Brazil; 6 Laboratoire Croissance, Réparation et Régénération Tissulaires (CRRET), CNRS ERL 9215, Université Paris Est Créteil, Université Paris Est, F-94000, Créteil, France

## Abstract

Synucleinophaties are progressive neurodegenerative disorders with no cure to date. An attractive strategy to tackle this problem is repurposing already tested safe drugs against novel targets. In this way, doxycycline prevents neurodegeneration in Parkinson models by modulating neuroinflammation. However, anti-inflammatory therapy *per se* is insufficient to account for neuroprotection. Herein we characterise novel targets of doxycycline describing the structural background supporting its effectiveness as a neuroprotector at subantibiotic doses. Our results show that doxycycline reshapes α-synuclein oligomers into off-pathway, high-molecular-weight species that do not evolve into fibrils. Off-pathway species present less hydrophobic surface than on-pathway oligomers and display different β-sheet structural arrangement. These structural changes affect the α-synuclein ability to destabilize biological membranes, cell viability, and formation of additional toxic species. Altogether, these mechanisms could act synergically giving novel targets for repurposing this drug.

The abnormal accumulation of α-synuclein amyloid aggregates in neural or glial cells is the common event in neurodegenerative disorders known as synucleinopathies^1^. It encompasses pathologies with devastating clinical prognosis like Parkinson’s disease (PD), dementia with Lewy bodies, and multiple system atrophy^2^,^3^,^4^. While the basis of neurotoxicity of α-synuclein remains controversial, it is now accepted that certain oligomeric species generated during α-synuclein amyloid self-assembly are most likely key players in the initiation^5^ and spreading of the pathology^6^. In addition, oxidative stress, mitochondrial dysfunction, and neuroinflammation have also been signed as responsible for neuronal cell death. However, compelling evidence shows that α-synuclein aggregation can trigger all these events^7^,^8^,^9^,^10^,^11^ and sometimes vice versa^12^, suggesting that these processes could be intertwined fostering a vicious circle with fatal consequences for neurons.

A significant number of molecules signed as efficient inhibitors of neuroinflammation, α-synuclein amyloid self-assembly, oxidative stress, or mitochondrial dysfunction *in vitro,* have failed to provide an effective neuro-protection *in vivo*^13^. Moreover, drugs developed to block one component of the death process at a time have proved ineffective in clinical trials and therefore nowadays the challenge must be directed in search of multi-targeted molecules to reach an efficient neuroprotective effect^14^. On the contrary, doxycycline, an antibiotic belonging to the family of tetracyclines, has been reported to exhibit neuroprotective activity in both MPTP^15^ and 6-OHDA^16^ animal models of PD. In fact, this antibiotic was able to mitigate the loss of dopaminergic neurons in the *substantia nigra pars compacta* and nerve terminals in the *striatum*. It has been proposed that the mechanism underlying this effect is the ability of doxycycline to diminish neuroinflammation^17^. Since treatment with anti-inflammatory compounds alone has not proved to be enough to prevent neurodegeneration^18^, we propose doxycycline to have an additional target. Indeed, doxycycline has been shown to inhibit fibril formation of amyloidogenic proteins such as Aβ peptide^19^, PrP peptide^20^ and β-microglobulin^21^. Nevertheless, to our knowledge, there are no mechanistic studies on the effect of doxycycline on either α-synuclein aggregation or seeding.

In the present paper, we show the ability of doxycycline to interfere with the pathologic cycle involved in synucleinopathies at the aggregation level for the first time. We demonstrate that doxycycline interacts with α-synuclein early aggregation intermediates leading to the formation of off-pathway species, with parallel β-sheet content, that do not evolve into fibril formation. In contrast to on-pathway oligomers, these aggregates are neither cytotoxic to dopaminergic cell lines, nor capable of disrupting the integrity of liposomes membrane. Furthermore, doxycycline is also able to block the seeding capacity of α-synuclein preformed aggregates. Taken together, our results shed light on the mechanism by which doxycycline affects α-synuclein aggregation and seeding of new oligomers. This novel mechanism of action might act in a synergic way with those previously proposed to explain the neuroprotective effect on models *in vivo*. Moreover, according to our data, subantibiotic doses of doxycycline would be high enough to interfere with the production of α-synuclein toxic species. Results presented herein place doxycycline as a pleiotropic drug becoming an attractive therapeutic strategy against synucleinopathies.

## Results

### Doxycycline inhibits α-synuclein aggregation through the formation of off-pathway oligomers

**Figure 1:**
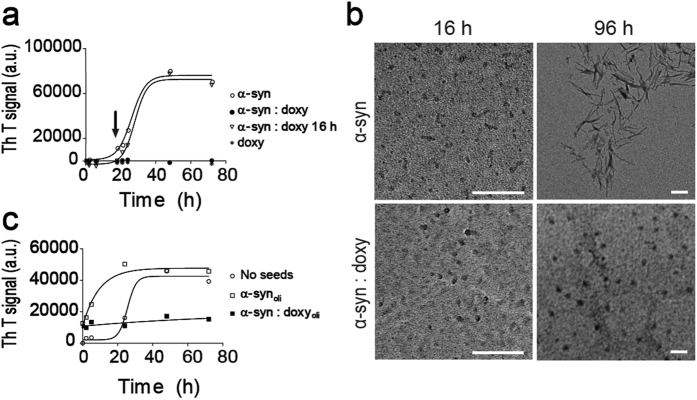
Effects of doxycycline on α-synuclein aggregation and seeding. (**a**) Fluorescence emission intensity of 25 μM thioflavin T in a solution containing 70 μM α-synuclein alone (empty circle), or with the addition of 100 μM doxycycline at 0 h (full circles), or after 16 h of incubation (inverted triangles) (doxycycline addition is indicated by an arrow). A solution containing 100 μM doxycycline alone (asterisk) is also shown as a control. (**b**) Transmission electron microscopy (TEM) of α-synuclein samples incubated at 37 °C under orbital agitation in the absence (top) or in the presence of doxycycline (bottom), and harvested after 16 h (left) or 96 h (right). The white bar corresponds to 200 nm at 220000X and 70000X magnification for observation of oligomers and fibrils respectively. (**c**) Fresh solutions of monomeric α-synuclein were seeded with oligomers preincubated in the absence (empty square) or in the presence of doxycycline (full square). The resulting solutions were incubated at 37 °C under orbital agitation and aggregation was assayed by ThT fluorescence emission. Unseeded aggregation kinetics are shown in (empty circle).

To analyze the ability of doxycycline to interfere with the fibril assembly process of α-synuclein, we incubated 70 μM α-synuclein in the absence or in the presence of 100 μM doxycycline at 37 °C under orbital agitation. Self-association kinetics of α-synuclein was monitored as an increase in fluorescence emission of Thioflavin T (ThT) at 482 nm (λ_exc_ 450 nm) upon binding of this probe to aggregates rich in cross-β structure^22^. In accordance to previous reports^23^, the aggregation kinetic of α-synuclein in the absence of doxycycline shows a lag phase of 18 h followed by an exponential growth to finally reach a plateau at 48 h (Fig. 1a). Conversely, in the presence of doxycycline the aggregation kinetic of α-synuclein is severely affected. According to the fluorescent probe, when doxycycline is added at the beginning of the incubation, the formation of aggregates with cross-β structure is dramatically diminished. However, once the exponential growth phase has been reached, addition of doxycycline has no observable effect on the aggregation kinetic of α-synuclein (Fig. 1a).

To evaluate the impact of doxycycline on the morphology of the species present along the aggregation pathway, we performed transmission electron microscopy (TEM) studies on samples of monomeric α-synuclein incubated with or without doxycycline and harvested at different times (Fig. 1b). After 16 h of incubation, we were able to detect oligomeric species in both conditions, which were morphologically undistinguishable by TEM. Nevertheless, only in the absence of doxycycline, oligomers evolved into fibrils, which were observed after 96 h incubation. This data suggests that doxycycline halt the assembly of α-synuclein oligomers into larger aggregates since only oligomers but not fibrils are still observed at this time. In fact, the absence of fibrils in the samples that contained doxycycline is consistent with the lack of cross-β observed using the ThT fluorescent probe (Fig. 1a).

To assess whether α-synuclein oligomers formed in the presence of doxycycline are on-pathway or off-pathway intermediates in the fibril formation process, we also carried out seeding experiments. For this, seeds were produced by incubating α-synuclein solution at 37 °C under orbital agitation for 16 h in the absence or presence of doxycycline. Then, an aliquot of the solution containing α-synuclein seeds was added to fresh solutions of monomeric α-synuclein and further incubated for 72 h (Fig. 1c). The addition of seeds formed in the absence of doxycycline accelerates the aggregation process and more species with cross-β structure are self-assembled from native α-synuclein as indicated by increased ThT fluorescence. In contrast, the oligomers formed in the presence of doxycycline are not able to further convert into amyloid fibrils. This indicates that oligomers formed in the presence of doxycycline did not function as template for the conversion of unpolymerized proteins into amyloid fibrils and we will further refer to them as off-pathway oligomers throughout the paper in contrast to the on-pathway oligomers formed in the absence of doxycycline.

### Inhibition of α-synuclein fibrillation is mediated by binding of doxycycline to oligomeric species

**Figure 2:**
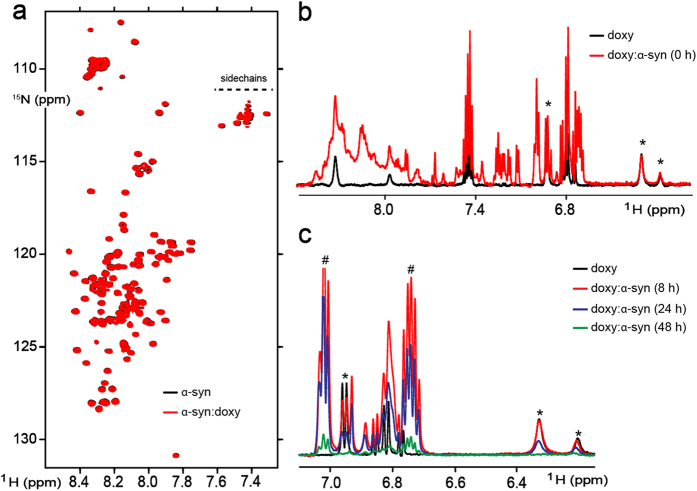
Analysis of doxycycline binding to α-synuclein by NMR. (**a**) Overlaid contour plots of ^1^H-^15^N SOFAST-HMQC spectra of 70 μM monomeric α-synuclein in the absence (black) or presence (red) of 350 μM doxycycline. (**b**) ^1^H NMR spectra of 200 μM doxycycline alone (black line) or upon the addition of 100 μM monomeric α-synuclein (red line). (**c**) ^1^H NMR spectra of 200 μM doxycycline in the absence (black line) or presence of 100 μM α-synuclein aged for 8 h (red line), 24 h (blue line) or 48 h (green line). In panels (**b**) and (**c**), the asterisk denotes isolated doxycycline ^1^H signals that broaden upon binding to higher order amyloidogenic structures of α-synuclein. In panel (**c**), the symbol # denotes isolated monomeric α-synuclein signals vanishing as a consequence of the progression of the aggregation process. NMR spectra were acquired at 15 °C (**a**) and 25 °C (**b**) and (**c**). Samples were dissolved in 20 mM HEPES supplemented with 150 mM NaCl and 10% D_2_O.

The anti-amyloid activity of doxycycline prompted us to explore details of its binding to α-synuclein by NMR spectroscopy. To analyze this interaction we used ^1^H-^15^N NMR heteronuclear multiple quantum correlation (HMQC) spectra. The ^1^H-^15^N spectrum of a sample of uniformly ^15^N-labeled α-synuclein, reflecting the intrinsically disorder nature of the protein, is shown in Fig. 2a. Upon titration of ^15^N-enriched α-synuclein with increasing concentrations of doxycycline, the ^1^H-^15^N HMQC spectra retained the excellent resolution of the uncomplexed, monomeric protein. Notably, no broadening or chemical shift perturbations could be observed, even at high ligand:α-synuclein ratios (5:1), indicating that doxycycline is unable to interact with monomeric α-synuclein.

To assess further the nature of the α-synuclein species involved in the interaction with doxycycline, we conducted NMR experiments aimed at detecting directly the signals belonging to the anti-amyloid compound. High-ordered oligomeric or prefibrillar α-synuclein species are invisible to the NMR approach used here^24^,^25^, although certain oligomers retaining high flexibility at the C-terminal region could be even detected^26^. Hence, a potential interaction of the antibiotic with those species will not become evident by using an NMR strategy based on the detection of protein backbone resonances. On the contrary, the 1D ^1^H NMR spectrum of doxycycline shows well-resolved resonances in the 6.0–7.0 ppm region, which constitutes an excellent probe for exploring the binding features of the antibiotic to α-synuclein. As expected, addition of 0.5 equiv. of monomeric α-synuclein to doxycycline samples caused no perturbations in the 1D ^1^H NMR spectrum of the small ligand, confirming the lack of interaction of the compound with the monomeric form of the protein (Figure 2b,c). By contrast, a substantial broadening of doxycycline signals was observed when aliquots of aged α-synuclein samples (24–48 h) were added, indicating that doxycycline is able to bind to the larger molecular species formed during the aggregation event (Fig. 2c). Even though changes in the signals of doxycycline upon the addition of α-synuclein aged for 8 h were not so prominent we still observed a small decrease in signal intensities, specially in the set of resonances centered at c.a. 6.95 ppm. These small perturbations are likely attributed to the small number of oligomers present in the sample at short aggregation times. In order to confirm this hypothesis, we analyzed the spectra recorded for doxycycline alone or upon the addition of fresh or aged (16 h) α-synuclein samples (Supplementary Figure S1). Although no fibrils could be detected on these samples (see Fig. 1a,d), they were centrifuged to spin down and remove minor insoluble species that could have been formed under our experimental conditions. Even after this treatment, signal attenuation in doxycycline remained unaltered (see the set of peaks centered at 6.95 ppm and 6.35 ppm), suggesting an interaction landscape formed mostly by soluble oligomers or early protofibrils. A comparative analysis between the intensities of α-synuclein resonances located at the methyl and aromatic regions (only signals showing no overlapping with doxycycline resonances were considered), shows no differences between the spectra recorded at 0 and 16 h, indicating the absence of slow-tumbling, highly-ordered structures such as amyloid fibrils or large protofibrils^27^, in full agreement with ThT and TEM data. Since monomeric α-synuclein is not able to interact with doxycycline (Fig. 2a,b and Supplementary Figure S1a), we assume that the attenuation observed for the signals of the compound under the experimental conditions described above are reflective of an interaction process with low-order oligomeric or protofibrillar α-synuclein species retaining some degrees of molecular flexibility. Altogether, these evidences indicate that binding of doxycycline to α-synuclein proceeds initially via interactions with aggregated species formed during the early stages of the assembly process, a molecular event that might provide a central mechanistic basis for the inhibitory process of doxycycline on α-synuclein fibrillation.

### Off-pathway oligomers present a different structural arrangement

In order to gain further insights on how doxycycline impacts on α-synuclein aggregation leading to the formation of off-pathway species, three complementary techniques were employed. These were small angle X-ray scattering (SAXS), bis-ANS fluorescence, and Fourier Transformed Infrared spectroscopy (FT-IR). SAXS reports changes in protein aggregates morphology (shape and size), bis-ANS infers the amount of hydrophobic surface exposed to the solvent, while FT-IR gives deeper insight into the oligomers structural organization being a technique particularly sensitive to β-sheet structure arrangements^28^.

**Figure 3:**
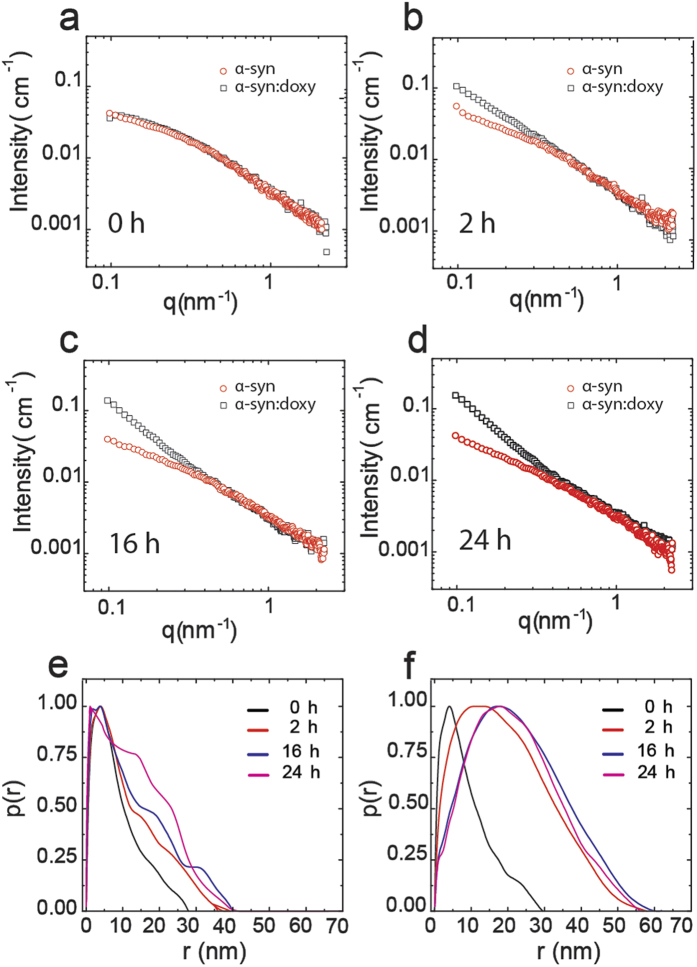
SAXS analysis of monomeric and oligomeric species of α-synuclein (**a**) SAXS curves from 175 μM α-synuclein incubated at 37 °C in the absence (red symbols) or presence (black symbols) of 250 μM doxycycline at 0 h (**a**), 2 h (**b**), 16 h (**c**) and 24 h (**d**). Corresponding distance distribution functions p(r) in the absence (**e**) or in the presence of doxycycline (**f**): 0 h (dark line); 2 h (red line), 16 h (blue line) and 24 h (magenta line).

For SAXS measurements, α-synuclein was incubated up to 24 h at 37 °C under orbital agitation in the absence or in the presence of doxycycline. SAXS data were acquired from aliquots taken at different elapsed times along the aggregation process (Fig. 3a–d). Note that right after its addition, doxycycline produces no immediate impact on the scattering curve from in-solution α-synuclein (Fig. 3a). However, its influence can be clearly detected after 2 h of incubation through the increase in the scattering intensity at low q values in comparison to the scattering from doxycycline-free α-synuclein (Fig. 3b). Such a behavior is a fingerprint of the formation of larger α-synuclein aggregates in comparison to those self-assembled in the absence of doxycycline. This was systematically reproduced over time. Therefore, SAXS data demonstrated that off-pathway α-synuclein oligomers formed in the presence of doxycycline are larger than on-pathway oligomers. Interestingly, the corresponding pair distance distribution functions, p(r), show that on-pathway oligomers evolve to fibril-like aggregates along time (Fig. 3e). The maximum frequency of distances (r_max_) inside the protein aggregate remains practically constant at 4 nm, while the maximum dimension, D_max_, elongates from 30 nm to 40 nm over 24 hours (Fig. 3e). On the other hand, off-pathway aggregates present a displacement toward larger distances in both r_max_ (up to 22 nm), and D_max_(up to 55 nm) (Fig. 3f). It should be stressed that aggregates larger than 55 nm can be formed but such dimension is over the upper detection limit of SAXS under our experimental conditions. Taken together with TEM results, the changes in the p(r) function features with time indicate that doxycycline impacts on α-synuclein aggregation pathway by deviating early elongated on-pathway oligomers into the formation of larger off-pathway oligomers (Fig. 1b).

**Figure 4:**
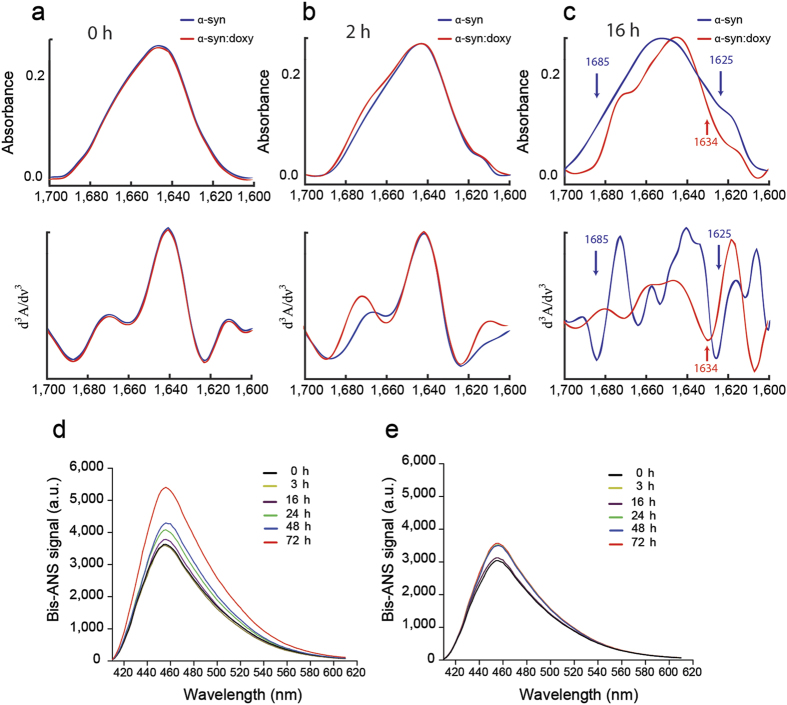
FTIR spectra of the amide I region of 280 μM α-synuclein in deuterated buffer in the absence (blue line) or in the presence (red line) of 400 μM doxycycline after 0 h (**a**), 2 h (**b**) and 16 h (**c**) incubation at 37 °C, pD 7 under orbital agitation. Deconvolved spectra (top) was performed using a mixed Gaussian/ Lorentzian line shape of 18 cm^−1^ and a resolution enhancement factor of 2. Derivative spectra (bottom) were obtained with a power of three and a break-point of 0.3. Bis-ANS fluorescence signal of α-synuclein solution (70 μM) incubated at 37 °C under orbital agitation in the absence (**d**) or in the presence of 100 μM doxycycline (**e**). Aliquots of the samples were taken throughout time. All results shown are representative of at least four independent experiments.

Infrared spectra, in the amide I region (1,700–1,600 cm^−1^), is particularly sensitive to the backbone conformation of proteins, allowing to discriminate the relative composition of secondary structure elements^29^. In order to perform a comparative analysis between α-synuclein on-pathway and off-pathway oligomers, the protein was incubated in the presence or in the absence of doxycycline and aliquots were taken at regular times for infrared spectra collection. At the beginning of incubation, samples are clearly very similar and suggest that the structural content is comparable regardless the presence of doxycycline (Fig. 4a). After Fourier self-deconvolution process, the amide I shows a contour centered at 1,649 cm^−1^ that is typical of an unfolded protein^30^. However, after 2 h incubation (Fig. 4b), off-pathways oligomers reflect increased content of ordered secondary structure, as revealed by the decrease in the FTIR band at 1,641 cm^−1^ (Table 1).

The half-width at half-height (HWHH) of the Amide I’ band reflects the structuration process of a protein and, as shown in Fig. 4b and c, off-pathway species seems to be more structured than than the on-pathway ones. Moreover, the amount of β-structure and the arrangement of the β-strands were also different in both species. In fact, off-pathway α-synuclein oligomers are rich in parallel β-sheet structure as shown by the presence of a band at 1,619–1,634 cm^−1^ and the absence of an absorption band at approximately 1,685 cm^−1^. On the contrary, the β-sheet structure of the on-pathway oligomers is predominantly antiparallel as indicated by a band at about 1,625 cm^−1^ with a high frequency component at approximately 1,685 cm^−1^, approximately fivefold weaker than the band at 1,625 cm^−1^. Remarkably, the differential organization of the β-sheet structure into parallel or antiparallel forms has been previously related to cytotoxicity^28^.

The intrinsic physicochemical properties of the oligomeric species, like hydrophobic solvent exposition, have also been associated to toxicity due to its relation to membrane binding and perturbation as well as cellular dysfunction. Accordingly, we measured the surface hydrophobicity of on-pathway and off-pathway α-synuclein oligomers using bis-ANS, a dye known to increase its fluorescence quantum yield upon binding to hydrophobic pockets on protein surfaces^31^. The emission spectra of bis-ANS when added to pre-incubated solutions of α-synuclein, reveals that there is a constant increase of hydrophobic surface exposure during aggregation (Fig. 4d), as reflected by a 65% enhancement in the quantum yield of the probe (calculated from areas under the curve). On the contrary, when doxycycline was present in the preincubated mixture, the fluorescence intensity remained unaltered up to 16 h (Fig. 4e), and reached a 10% increase in fluorescence between 24 and 72 h, indicating that off-pathway oligomers expose hydrophobic patches to a lesser extent.

Overall, these results suggest that doxycycline induces differential conformational changes in α-synuclein oligomers, resulting in the formation of off-pathway oligomers with well-distinctive features in terms of shape, size, structure and hydrophobic surface exposure.

### Doxycycline reshapes α-synuclein into non-toxic oligomers

**Figure 5:**
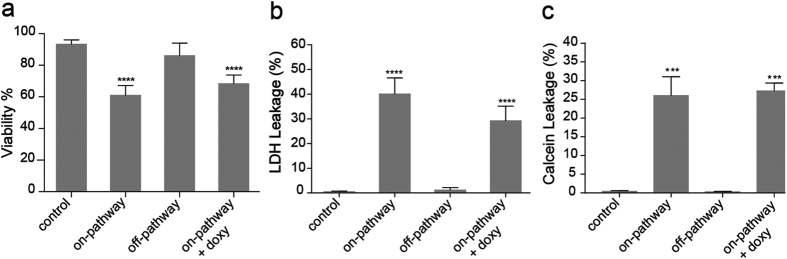
Modulation of α-synuclein oligomers cytotoxicity by doxycycline. (**a**) Cell viability, and (**b**) cell death of SH-SY5Y cells 24 h after the addition of 20 mM HEPES pH 7.4, 150 mM NaCl buffer (control), on-pathway α-synuclein oligomers, off-pathway α-synuclein oligomers, or on-pathway α-synuclein oligomers with doxycycline added to the culture medium. (**c**) Changes in liposomal membrane permeability upon the addition of the same species. The fluorescence signal was normalized by the signal observed after the addition of Triton X-100, which induced complete disruption of the vesicles. A monomeric solution of α-synuclein was also used as a negative control for cell viability, cytotoxicity and leakage assays. On-pathway, and off-pathway α-synuclein oligomers were prepared by incubating 140 μM α-synuclein in the absence, or in the presence of 200 μM doxycycline respectively for 16 h under orbital agitation at 37 °C.

Cytotoxicity of α-synuclein has been attributed to transient prefibrillar species formed during protein aggregation^5^. Indeed, the pathogenicity associated to the these prefibrillar species has been linked to the conformational arrangement of the aggregate species as revealed by conformation-specific antibodies^32^ as well as FTIR studies^28^. Therefore, we assessed the impact of the structural changes induced by doxycycline on the pathogenicity of α-synuclein oligomers. First, we carried out cell viability studies on SH-SY5Y cell line using the MTT assay^33^, which reports viable cell number based on the mitochondrial activity. For this, on-pathway and off-pathway α-synuclein oligomers were obtained by preincubation of 140 μM α-synuclein in the absence or presence of 200 μM doxycycline, respectively for 16 h at 37 °C under orbital agitation. A 25 μl aliquot of each preparation was added to SH-SY5Y cells and further incubated at 37 °C. As previously described^23^, on-pathway oligomeric species were able to decrease cell viability as reflected by the 40% decrease in the turnover of MTT (Fig. 5a). When cells were treated with off-pathway oligomers we observed no significant difference from controls on MTT turnover. It should be noticed that the presence of doxycycline in the culture medium was not capable to prevent the deleterious effect that on-pathway oligomers exerted on SH-SY5Y cells.

Interestingly, one of the proposed mechanisms by which α-synuclein oligomers produce cytotoxicity is by inducing a perturbation on the cell membrane integrity^34^. Therefore, we measured the release of the cytosolic enzyme lactate dehydrogenase (LDH) from cells into the medium, which is commonly used as a measure of cytotoxicity by membrane impairment (Fig. 5b). Once again, on-pathway oligomers showed cytotoxicity against SH-SY5Y cells as reflected by the 40% increase in the release of LDH into the culture medium. On the contrary, in the presence of off-pathway oligomers cell integrity is well preserved. Preservation of cell integrity is not observed when doxycycline is added to the culture medium before the addition of on-pathway oligomers.

The differential capability of α-synuclein oligomers to affect membrane integrity, as showed by LDH assays, was also analyzed by content leakage assays on synthetic membranes. To do that, we monitored the release of calcein entrapped in rat brain lipid vesicles upon the addition of α-synuclein oligomers (Fig. 5c). In line with cytotoxicity assays, on-pathway α-synuclein oligomers induced the release of the fluorescent probe from liposomes, confirming the ability of these species to disrupt membrane integrity; while off-pathway oligomers showed no destabilizing effect over lipid membranes. The addition of doxycycline to liposomes did not prevent calcein release induced by on-pathway oligomers showing that the antibiotic does not stabilize the membrane by a direct interaction nor does it blocks the deleterious effect of on-pathway oligomers upon binding to their surface.

Altogether, these results suggest that the protective effect of doxycycline could be attributed to the remodeling of α-synuclein into off-pathway oligomers that are not able to impair membrane integrity and not by a direct effect of the antibiotic on the cells.

## Discussion

The current consensus suggests that synucleinophaties develop from complex gene–environment interactions and, thus, more than one causative process is underlying the neurodegenerative pathogenesis. In fact, protein aggregation, neuroinflammation, oxidative stress, mitochondrial functional disruption, and lysosomal dysfunction constitute an endless loop with fatal consequences on neurons.

In spite of the long-standing search for new drugs aimed at reducing the impact of synucleinophaties and particularly of PD, nowadays the available therapies for these pathologies are merely palliative. Indeed, drugs that are able to block the process *in vitro* do not work properly *in vivo* due to their toxicity or low bioavailability in the brain. In order to overcome this failure, alternative drug development strategies are now being explored, such as the repurposing of existing compounds with novel targets^35^ *i.e.* take advantage of the treasure of potential therapeutics in the pharmacopoeia. This could save a considerable amount of time and money since information about the pharmacokinetics, pharmacodynamics and drug side effects are already established.

Doxycycline, a semi-synthetic second-generation tetracycline, is used currently as a typical antibiotic in humans having clinical safety record and good penetration of the blood–brain barrier^36^. In addition to its well-characterised antibiotic effect, doxycycline has revealed a range of different targets mainly focused on the regulatory influence on the immune system, inflammatory pathway and oxidative stress^37^,^38^. In fact, by tuning the doxycycline concentration, it is possible to select the anti-inflammatory or antibiotic activity. In this way, some clinical trials demonstrated that antibiotic dose administration (200–400 mg/day) is responsible for the antimicrobial effect which may lead to develop bacterial resistance and endogenous flora alterations, whereas subantibiotic doses (20–40 mg/day) do not alter bacteria susceptibility to antibiotics and exert anti-inflammatory activities^39^. Neuroprotective properties of doxycycline in models of cerebral ischemia, spinal cord injury, PD, Huntington’s disease, amyotrophic lateral sclerosis and multiple sclerosis were also reported^40^,^41^,^42^. The anti-inflammatory properties of doxycycline have been proposed as the mechanism involved in the neuroprotective effect. Nevertheless, treatment with conventional anti-inflammatory drugs has not proved effective in neuroprotection, suggesting that this mechanism alone would not be enough^18^.

Here we demonstrate an additional mechanism by which doxycycline could exert neuroprotection which could complement the previously well-reported anti-inflammatory^17^,^39^ and anti-oxidant effects^38^. We show for the first time that the presence of doxycycline induces a remodelling of α-synuclein oligomers into off-pathway non-toxic, non-seeding species. This remodelling process is only efficient on early species of the aggregation process, since the addition of doxycycline after 16 h incubation did not produce any change in the α-synuclein aggregation kinetics or its cytotoxicity (Fig. 1a and b). According with NMR data, doxycycline is able to bind oligomeric α-synuclein species, but not to the monomeric protein. Congo Red, lacmoid and epigallocatechin gallate, are also small molecules which inhibit α-synuclein fibrillization by interacting with the monomeric protein^26^,^43^. On the contrary, doxycycline binds only to oligomeric species preserving the monomeric form of the protein for its physiological function^26^.

Electron microscopy showed that doxycycline-remodelled oligomers do not evolve to fibrils. In addition, fluorescence studies show that the formation of cross-β structure is halted in the presence of doxycycline. Moreover, lesser hydrophobic surface was detected, suggesting a different structural arrangement of the intermediate species. In fact, infrared studies demonstrated the presence of parallel β-sheet instead of the typical antiparallel β-sheet reported for on-pathway toxic oligomeric species^28^. The impact of doxycycline on the assembly of α-synuclein oligomers is also evidenced by the changes in size and shape of the aggregates, as revealed by SAXS experiments. Overall, the different structural organization of off-pathway oligomers results on a decreased ability to alter cell viability and membrane permeability, as compared to on-pathway oligomers.

Baicalein is an active flavonoid, which also induces the formation of non-toxic off-pathways α-synuclein oligomers, but its use as neuroprotector is controversial since the flavonoid triggers intrinsic and extrinsic apoptotic pathways^44^. In the same way, gallic acid (3,4,5-trihydroxybenzoic acid) and some of its derivatives may interact with α-synuclein oligomers by a mechanism similar to doxycycline, *i.e.* halting the fibrillation process through the formation of off-pathway non-toxic oligomers which are unable to induce the seeding on monomeric α-synuclein^45^. However, there is no data on bioavailability or pharmacokinetics or side effects of this compound *in vivo*.

Curcumin (diferuloylmethane) also reduces significantly cell toxicity of α-Syn aggregates by binding to preformed oligomers and fibrils^46^ with effectiveness comparable with doxycycline (1:1 molar ratio)^47^. However, the instability, low solubility, little oral bioavailability of curcumin limit its clinical applications and to overcome this drawback, some structural curcumin analogues with antiaggregation properties are been developed. In this context, no human long-term toxicity studies have been reported until now with curcumin structural analogues^48^. On the contrary, oral administration of doxycycline proved to be a safe and effective drug for dermatologically long-term treatments^49^,^50^. Moreover, it was also reported that sub-antibiotic doses of doxycycline have no effect on the composition or antibiotic resistance of different microflora^51^.

**Figure 6:**
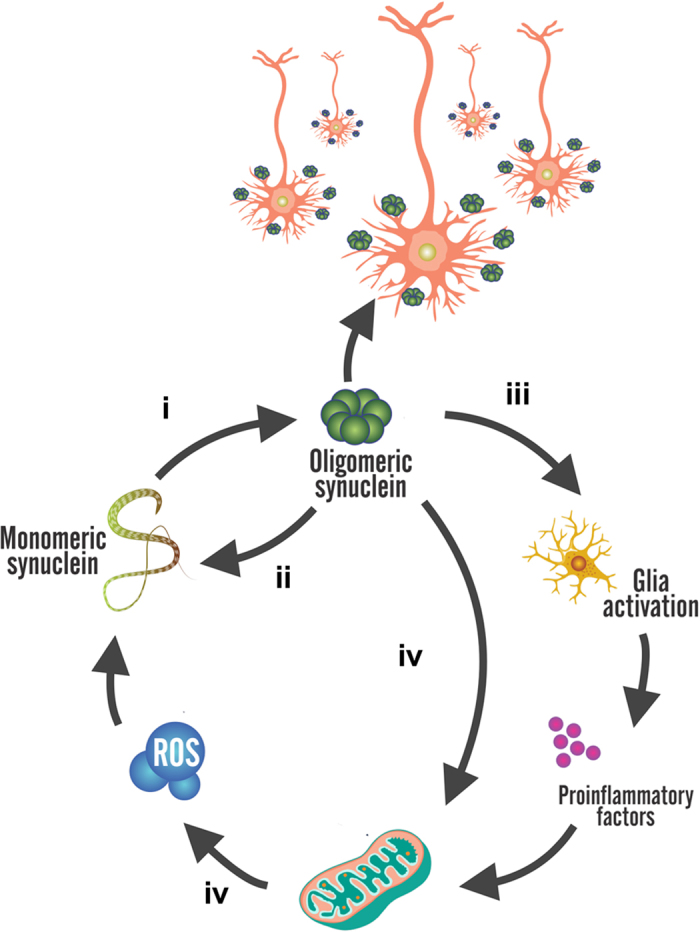
Schematic diagram of the feedback relationship between α-synuclein, mitochondria and glial cells. The oligomeric species of α-synuclein provoke mitochondria fragmentation and ROS production is increased. ROS impacts on monomeric α-synuclein triggering its aggregation. The oligomeric α-synuclein released to the extracellular matrix inducing glia activation. Liberation of pro-inflammatory factors induces mitochondrial damage in an endless cycle. Doxycycline can interfere with this vicious circle blocking (**i**) α-synuclein oligomers nucleation and (**ii**) seeding, (**iii**) glia activation^16^, and (**iv**) ROS activity^37^.

The seeding effect on monomeric α-synuclein, characteristic of toxic oligomers, was also blocked in the presence of doxycycline. It has been shown that toxic species of α-synuclein may be transmitted from diseased cells to healthy neurons where they induce the conversion of native α-synuclein into toxic oligomeric species, following a seeding-nucleation mechanism^52^,^53^. Our results suggest that doxycycline structurally remodelled species abolish the seeding process and, thus, they would interfere with the spreading process (Fig. 6).

Putting together our results, the presence of doxycycline can attenuate the pathological cycle described in Fig. 6 at five different points: inhibiting synuclein aggregation and seeding, neuroinflammation in glial cells, mitochondrial dysfunction and ROS damage.

According to our dose:response studies (see Supplementary Figure S2), an equimolar concentration of doxycycline to α-synuclein would be suitable to reach a protective effect. While in cerebrospinal fluid the concentration achieved by doxycycline during the oral treatment as antimicrobial (200 mg twice day) is about 3 μM (considering the main penetration of the antibiotic in 26%) the α-synuclein concentration is about 0.12 nM^54^. This data strongly suggests that doxycycline in subantibiotic doses (20–40 mg/day) would be enough to exert neuroprotection.

The results presented herein reveal potential protective side effects for doxycycline in the pathogenesis cycle of synucleinopathies that could be exploited repurposing an old safe drug.

## Methods

### Preparation of α-synuclein

Expression and purification of recombinant human α-synuclein was performed as previously described^55^. The purity of the protein was assessed by SDS-PAGE. Monomeric α-synuclein stock solutions were prepared in 20 mM HEPES, 150 mM NaCl, pH 7.4. Prior to measurements, protein solutions were filtered and centrifuged for 30 min at 12,000 × *g*. Protein concentration was determined by the measurement of absorbance at 280 nm using extinction coefficient ε_275_ = 5600 cm^−1^ M^−1^.

### Protein aggregation

The aggregation protocol was adapted from previous studies^56^. Monomeric α-synuclein solutions (70 μM) in 20 mM HEPES, 150 mM NaCl, pH 7.4, were incubated in a Thermomixer comfort (Eppendorf) at 37 °C under orbital agitation at 700 rev./min in the absence or in the presence of 100 μM doxycycline.

### Thioflavin T assay

Formation of cross-β structure during aggregation was followed by addition of Thioflavin T (ThT) fluorescent probe on aliquots withdrawn from the incubation mixture at different times, according to LeVine^22^. Changes in the emission fluorescence spectra with the excitation wavelength set at 450 nm was monitored using an ISS (Champaign, IL) PC1 spectro fluorometer.

### Transmission Electron Microscopy

Samples (30 μl) of a 14 μM α-synuclein solution were adsorbed onto glow-discharged 200 mesh formvar carbon coated copper grids (Electron Microscopy Sciences) and stained with aqueous uranyLess (Electron Microscopy Sciences). Excess liquid was removed and grids were allowed to air dry. Samples were viewed using a Hitachi 7700 transmission electron microscope.

### Bis-ANS

α-synuclein was aggregated as described above in the absence or presence of doxycycline. Aliquots were taken at regular time and bis-ANS was added to a final concentration of 5 μM. Bis-ANS was excited at 395 nm, and fluorescence emission was collected from 410 to 610 nm.

### SAXS

Small Angle X-ray Scattering (SAXS) data from 175 μM α-synuclein, in the absence or presence of doxycycline were obtained on SAXS1 beamline at the *Laboratório Nacional de Luz Síncrotron* (LNLS, Campinas, Brazil). The radiation wavelength was set to 0.148 nm and a Pilatus 300 detector was used to record the scattering patterns. The sample-to-detector distance was set to ~1000 mm allowing us to explore a minimum scattering vector of 0.10 nm^−1^, where *q* is the magnitude of the scattering *q*-vector defined by *q* = (4π/*λ*)sin*θ* (being *2θ* the scattering angle). In this way, the maximum particle distance accessible from our experimental set-up was *circa* 60 nm. Samples were set between two mica windows and a 1 mm spacer, handled in a liquid sample-holder placed perpendicular to the incoming beam. The obtained curves were normalized by taking into account the X-ray beam intensity decrease during the experiment. The scattering curve of the buffer solution was subtracted from the sample’s SAXS curves, considering each sample’s attenuation. All measurements were taken at room temperature.

A Fourier Transform connects the scattering intensity, I(q), to the pair distance distribution function p(r) in the absence of interference effects. Such a function is model-free and represents the frequency of distances within the entire volume of the scattering particle, thus giving information to the particle maximum dimension, D_max_, where p(r) goes to zero. In the current paper, p(r) functions from SAXS curves were obtained by making use of GNOM software^57^.

### NMR

All NMR spectra were recorded on a Bruker 600 MHz Avance III spectrometer using a cryogenically cooled triple resonance ^1^H(^13^C/^15^N) TCI probe. 1D ^1^H and 2D ^1^H-^15^N SOFAST-HMQC (band-Selective Optimized Flip Angle Short Transient) experiments were recorded at 15 °C on samples dissolved in buffer Hepes 20 mM at pH 7.4 in the presence of 150 mM NaCl and 10% D_2_O. SOFAST-HMQC spectra were acquired with the following parameters: 32 scans, 30 ms recycling delay, 1 K and 256 increments and SW of 16 and 26 ppm in the ^1^H and ^15^N dimension, respectively. After acquisition, spectra were zero-filled to 4 K (^1^H) and 2 K (^15^N), processed with sine bell apodization and baseline corrected.

To characterise the interaction between doxycycline and aged α-synuclein we incubated solutions of monomeric α-synuclein (300 μL, 100 μM) at 37 °C under constant stirring at 280 rpm using small magnetic bars. Samples were dissolved in 20 mM Hepes buffer at pH 7.4 with 150 mM NaCl and 10% D_2_O. The formation of amyloid fibrils was monitored by drawing 2 μL aliquots at different times from each sample, diluting them into 2 mL solutions in the presence of 10 μM thioflavin T and measuring its fluorescence at 480 nm as previously described^56^. We removed the samples from the incubator at times 0, 8, 24 and 48 h, spiked them with 200 μM doxycycline from a 5 mM freshly prepared stock solution and immediately run the 1D-^1^H NMR spectra. Thioflavin-T fluorescence plateau after ~40 h of incubation under these aggregation conditions. In another set of experiments, samples of α-synuclein aged for 0 h and 16 h were centrifuged for 1 h, at 15000 rpm, 4 °C to remove insoluble species that might have formed under the experimental conditions of our experiments, before adding them to the doxycycline solution. The NMR spectra were recorded using 8 K points, a SW of 16 ppm, 512 scans and a recycling delay of 1 sec. Spectra were zerofilled to 32 K, processed with sine bell apodization and baseline corrected. All spectra were acquired and processed using Topspin 3.2 (Bruker) and analyzed using Topspin (1D ^1^H) and Sparky (^1^H-^15^N SOFAST-HMQC).

### Infrared Spectroscopy

Samples at 280 μM of α-synuclein in the absence or the presence of 400 μM doxycycline in buffer 20 mM HEPES, 150 mM NaCl, pD 7.0, D_2_O were collected after 0, 2 and 16 h of orbital incubation at 37 °C. Since monomeric α-synuclein was higher than the oligomeric species, after incubation we partially separated from the monomer using an Amicon Ultra-0.5 100 kDa cut-off filter and assembled in a thermostated cell between two CaF_2_ windows with a path length of 50 nm. The spectra were recorded in a Nicolet 5700 spectrometer equipped with a DTGS detector (Thermo Nicolet, Madison, WI) as previously described^29^. The sample chamber was permanently purged with dry air. The spectra were generated by averaging 256 interferograms collected with a nominal resolution of 2 cm^−1^ and apodized with a Happ-Genzel function. The D_2_O contribution in the amide I´ region was eliminated by subtracting the buffer spectra from that of the solution at the same temperature to obtain a flat baseline between 1,900 and 1,700 cm^−1^. Solvent subtraction, deconvolution, determination of band position and curve fitting of the original amide I band were performed as described^29^. The error in determination of the FTIR structural analysis from the amide I band from different runs is 3%.

### Human neuroblastoma cell culture

SH-SY5Y cells were grown in DMEM supplemented with 10% fetal bovine serum (FBS) and 1% penicillin/streptomycin (PS), at 37 °C and 5% CO_2_. For cell viability and cytotoxicity assay cells were seeded in 96 wells plates at 15,000 cells/well, and 30,000 cells/well, respectively and maintained in 100 μl of DMEM supplemented with 10% FBS and 1% PS for 24 h at 37 °C. Afterwards, cells were treated with a 25 μl aliquot of 20 mM HEPES, 150 mM NaCl buffer, pH 7.4 (control), or 140 μM α-synuclein aged for 16 h at 37 °C under orbital agitation in the absence (on-pathway oligomers) or presence of 200 μM doxycycline (off-pathway oligomers). Alternatively, on-pathway oligomers were also added together with doxycycline to the culture medium (on-pathway oligomers +doxy). After treatment, cells were incubated for 24 h at 37 °C 5% CO_2_. To determine cell viability, the colorimetric MTT metabolic activity assay was used as previously described by Mosmann^33^. All experiments were performed in sextuplicate, and the relative cell viability (%) was expressed as a percentage relative to the untreated cell control. Cytotoxicity was determined using the Cytotoxicity Detection Kit (Roche) following instructions from the manufacturer. The assay is based on the measurement of the activity of lactate dehydrogenase (LDH) released into the culture medium upon permeabilization or lysis of cells. Percentage of cytotoxicity is referred to the LDH activity released from cells after treatment with 1% Triton X-100.

### Calcein Release Assay

The lipid mixture used for this experiment was extracted from brain membranes of Wistar white rats by Folch method^58^. Rats were provided by the “Bioterio” Instituto de Química Biológica (Facultad de Bioquímica, Química y Farmacia, UNT, Tucumán. Argentina). Rats were obtained by exocria and maintained under controlled temperature and humidity under a 12 h light-dark cycle. Animals were maintained and treated in accordance with the criteria established in the “Guide for the care and use of laboratory animals”, published by the “Institute of Laboratory Animal and National Research (1999)”. After extraction, lipids were stored in chloroform:methanol (2:1, v/v). For the preparation of large multilamellar vesicles (MLVs), lipids were dried under nitrogen onto the wall of a Corex glass tube, placed in a vacuum oven to completely remove any remaining solvent and then rehydrated in 25 mM Tris, 50 mM calcein, pH 7.4 buffer. In order to obtain small unilamellar vesicles (SUVs), we followed the protocol described by Finer^59^. Briefly, MLVs were sonicated with probe-type sonicator under nitrogen and controlled temperature. To remove titanium debris the suspension was centrifuged for 15 min at 1100 × g. In order to separate Calcein- loaded SUV from free dye we used Sephadex G-75. During incubation, changes in the fluorescent intensity of the different mixtures were monitored at λ_exc_ = 490 nm, λ_em_ = 510 nm in a ISS (Champaign, IL, USA) PC1 spectrofluorometer as previously described by Kendall (47). α-synuclein (140 μM) incubated in the absence (on-pathway oligomers) or in the presence of 200 μM doxycycline (off-pathway oligomers) during 16 h were added to 50 μM of lipid vesicles. Total dye release was completed by the addition of 0.1 vol % Triton X-100. The percentage of probe release was calculated as follows:


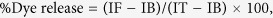


where IF, IT, and IB are the fluorescence intensity of the dye released by the protein, total dye released, and control blank.

## Additional Information

**How to cite this article**: González-Lizárraga, F. *et al*. Repurposing doxycycline for synucleinopathies: remodelling of α-synuclein oligomers towards non-toxic parallel beta-sheet structured species. *Sci. Rep.* **7**, 41755; doi: 10.1038/srep41755 (2017).

**Publisher's note:** Springer Nature remains neutral with regard to jurisdictional claims in published maps and institutional affiliations.

## Supplementary Material

Supplementary Information

## Figures and Tables

**Table 1:** FTIR-based evaluation of secondary structure content during the fibrillation process for human α-synuclein with doxycycline introduced at selected time.

	Band position (cm^−1^)	% Area	Assignment
α-synuclein or α-synuclein:doxycycline (0 h)	1,625	10	Extended
	1,642	44	Disorder/Extended
	1,657	27	Loops/Disorder
	1,671	19	β-turn
	1,682	<1	—
α-synuclein (2 h)	1,625	13	Extended/β-Sheet
	1,641	40	Disorder/Extended
	1,657	32	Loops/Disorder
	1,671	11	β-turn
	1,685	4	β-Sheet (High component)
α-synuclein:doxycycline (2 h)	1,629	24	Extended/β-Sheet
	1,641	27	Disorder/Extended
	1,656	30	Loops/Disorder
	1,671	18	β-turn
	1,680	<1	—
α-synuclein (16 h)	1,625	13	Antiparallel β-Sheet
	1,640	31	Disorder/Extended
	1,656	33	Loops/Disorder
	1,672	19	β-turn
	1,687	4	β-Sheet (high freq.component)
α-synuclein:doxycycline (16 h)	1,619	6	Extended/Side chains
	1,634	21	β-Sheet
	1,644	32	Disorder/Extended
	1,657	24	Loops
	1,671	22	β-turn
	1,684	<1	—
