# Supplementary material for: Repurposing doxycycline for synucleinopathies: remodelling of α-synuclein oligomers towards non-toxic parallel beta-sheet structured species
Source: Sci Rep. 2017 Feb 3;7:41755. doi: 10.1038/srep41755 (PMC5290535; doi:10.1038/srep41755)
Supplement: Supplementary Information [file srep41755-s1.pdf]

Supplementary Information for:

Repurposing doxycycline for synucleinopathies: remodelling of α-synuclein oligomers towards non-toxic parallel beta-sheet structured species

Florencia González-Lizárraga[1,2], Sergio B. Socías[1], César L. Ávila[1], Clarisa M. Torres-Bugeau[1], Leandro R. S. Barbosa[3], Andres Binolfi[4], Julia E. Sepúlveda Díaz[2], Elaine Del-Bel[5], Claudio O. Fernandez[4], Dulce Papy-Garcia[6], Rosangela Itri[3], Rita Raisman-Vozari[2], and Rosana N. Chehín*[1]*

[1]Instituto Superior de Investigaciones Biológicas (INSIBIO), CCT-Tucumán and Instituto de Química Biológica Dr Bernabé Bloj (CONICET-UNT) Chacabuco 461 (T4000ILI) Tucumán, Argentina.

[2]Sorbonne Universite, UPMC Univ Paris 06, INSERM, CNRS, UM75, U1127, UMR 7225, Institut du Cerveau et de la Moelle Epinière, Paris, France.

[3]Instituto de Física da Universidade de São Paulo – IFUSP, Rua do Matão, Travessa R, 187, São Paulo, Brazil.

[4]Max Planck Laboratory for Structural Biology, Chemistry and Molecular Biophysics of Rosario (MPLbioR, UNR-MPIbpC) and Instituto de Investigaciones para el Descubrimiento de Fármacos de Rosario (IIDEFAR, UNR-CONICET), Universidad Nacional de Rosario , Ocampo y Esmeralda, S2002LRK Rosario, Argentina.

[5]Department of Morphology, Physiology and Stomatology, Faculty of Odontology of Ribeirão Preto, University of São Paulo, Brazil; Center of Interdisciplinary Research on Applied Neurosciences (NAPNA), University of São Paulo, Brazil.

[6]Laboratoire Croissance, Réparation et Régénération Tissulaires (CRRET), CNRS ERL 9215, Université Paris Est Créteil, Université Paris Est, F-94000, Créteil, France.

* Correspondence must be addressed to RCH (rosanachehin@gmail.com) or RRV (ritaraisman@gmail.com)

1. Analysis of doxycycline binding to α-synuclein by NMR

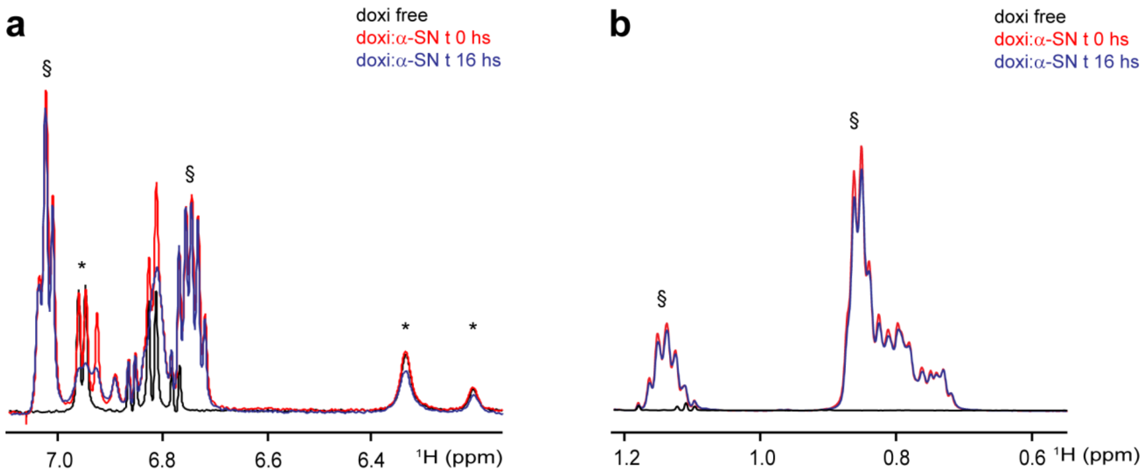

Figure S1. Analysis of doxycycline binding to α-synuclein by NMR. ^{1}H 1D NMR spectra in the aromatic **(a)** and methyl region **(b)** of 200 µM doxycycline alone (black line) and upon addition of 100 µM monomeric α-synuclein (red line) or α-synuclein that was aged for 16 hs (blue line). Before preparing the mixtures, the α-synuclein samples were spun down to remove insoluble particles that might have formed during this time frame. Asteriks denote isolated doxycycline ^{1}H NMR signals that broaden upon binding to α-synuclein oligomers or protofibrils. The symbols § indicate isolated clusters of α-synuclein ^{1}H NMR signals. We did not detect major changes in α-synuclein signals between time 0 and 16 hs indicating that slow-tumbling, highly ordered structures such as amyloid fibrils or large protofibrils were not formed at great extents. NMR spectra were acquired at 25°C. Samples were dissolved in 20 mM HEPES supplemented with 150 mM NaCl and 10% D_2O.

2. Dose:response effect of doxycycline on α-synuclein aggregation

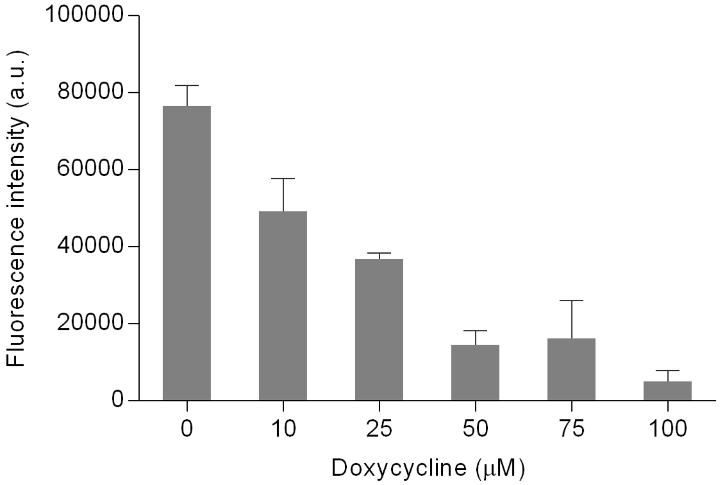

Figure S2. Dose-response effect of doxycycline on the α-synuclein fibrillation process. A solution containing 70 μM α-synuclein in 20 mM HEPES buffer, 150 mM NaCl, pH 7.4 was incubated at 37ºC under orbital agitation for 48 h in the presence of 0 μM, 10 μM, 25 μM, 50 μM, 75 μM and 100 μM doxycycline as described in Methods. On each sample Thioflavin T was added to a final concentration of 25 μM and the formation of cross-beta structure was estimated by the increase in fluorescence at λ_{exc}=450 nm, λ_{em}=482 nm.
